# Fully recombinant IgG2a Fc multimers (stradomers) effectively treat collagen-induced arthritis and prevent idiopathic thrombocytopenic purpura in mice

**DOI:** 10.1186/ar4024

**Published:** 2012-08-20

**Authors:** Ajay Jain, Henrik S Olsen, Ravi Vyzasatya, Erin Burch, Yukimi Sakoda, Emmanuel Y Mérigeon, Ling Cai, Changwan Lu, Ming Tan, Koji Tamada, Dan Schulze, David S Block, Scott E Strome

**Affiliations:** 1 Division of General and Oncologic Surgery, University of Maryland School of Medicine, 22 South Greene Street, Room S4B12, Baltimore, MD 21201, USA; 2 Department of Otorhinolaryngology-Head and Neck Surgery, University of Maryland School of Medicine, 16 S. Eutaw Street, Baltimore, MD 21201, USA; 3 Baltimore Veterans Administration Medical Center, 10 N. Greene Street, 5C Surgical Services Area, Baltimore, MD 21201, USA; 4 Gliknik Inc., 801 West Baltimore Street, Suite 501A, Baltimore, MD 21201, USA; 5 Division of Biostatistics & Bioinformatics, Department of Epidemiology and Public Health & University of Maryland Greenebaum Cancer Center, University of Maryland School of Medicine, 660 W. Redwood Street, Suite 109, Baltimore, MD 21201, USA; 6 Department of Microbiology and Immunology, University of Maryland School of Medicine, 685 West Baltimore Street, Suite 385, Baltimore, MD 21201, USA

## Abstract

**Introduction:**

Soluble immune aggregates bearing intact Fc fragments are effective treatment for a variety of autoimmune disorders in mice. The better to understand the mechanisms by which Fc-bearing immune complexes suppress autoimmunity, and to develop a platform for clinical translation, we created a series of fully recombinant forms of polyvalent IgG2a Fc, termed stradomers, and tested their efficacy in a therapeutic model of collagen-induced arthritis (CIA) and preventive models of both idiopathic thrombocytopenic purpura (ITP) and graft-versus-host disease (GVHD).

**Methods:**

Stradomers were created by engineering either the human IgG2 hinge sequence (IgG2H) or the isoleucine zipper (ILZ) onto either the carboxy or amino termini of murine IgG2a Fc. Multimerization and binding to the canonical Fc receptors and the C-type lectin SIGN-RI were evaluated by using sodium dodecylsulfate-polymerase chain reaction (SDS-PAGE) and Biacore/Octet assays. The efficacy of stradomers in alleviating CIA and preventing ITP and GVHD was compared with "gold standard" therapies, including prednisolone and intravenous immune globulin (IVIG).

**Results:**

Stradomers exist as both homodimeric and highly ordered sequential multimers. Higher-order multimers demonstrate increasingly stable associations with the canonic Fcγ receptors (FcγRs), and SIGN-R1, and are more effective than Fc homodimers in treating CIA. Furthermore, stradomers confer partial protection against platelet loss in a murine model ITP, but do not prevent GVHD.

**Conclusion:**

These data suggest that fully human stradomers might serve as valuable tools for the treatment of selected autoimmune disorders and as reagents to study the function of Fc:FcR interactions *in vivo*.

## Introduction

Soluble immune complexes containing IgG1 Fc can suppress ongoing autoimmunity in a variety of animal models [1]. For example, OVA-anti-OVA conjugates, aggregated intravenous immunoglobulin (IVIG) products, and antibody-coated liposomes are therapeutically efficacious in murine models of idiopathic thrombocytopenic purpura (ITP) and rheumatoid arthritis [2-5]. The translational relevance of these animal findings is supported by clinical studies in ITP, in which the therapeutic effects of IVIG directly correlate with the presence of immune aggregates in the sera [6], and intramuscular (IM) administration of anti-D immunoglobulin, containing a high aggregate percentage, improves platelet counts even in Rh-negative patients [7]. Despite data supporting the therapeutic potential of IgG containing Fc multimers, to the best of our knowledge, no one has generated and evaluated the antiinflammatory and antiautoimmune properties of fully recombinant IgG Fc multimers for potential clinical application.

As a precursor to the development of fully recombinant human IgG1 containing Fc multimers for clinical use, we sought to identify naturally occurring "multimerization domains" (MDs) with limited immunogenic potential. In serum, human IgG2 naturally exists as both monomers (homodimers) and low-abundance dimers, with dimer formation recognized to depend on the hinge region [8-10]. In this report, we demonstrate that fully recombinant fusion proteins composed of murine IgG2a Fc and either the human IgG2H or isoleucine zipper (ILZ) MDs exist as both monomers (homodimers) and as a surprisingly high percentage of covalently linked highly ordered multimers. We called these Fc multimers stradomers to distinguish them from the naturally occurring homodimeric form of intact immunoglobulins. These novel fusion proteins can alleviate collagen-induced arthritis (CIA) and prevent ITP in murine models, raising the possibility that fully human variants may have therapeutic potential for the treatment of a variety of rheumatologic diseases [9,10].

## Materials and methods

### Mice

We used 6- to 8-week-old, female C57BL/6 mice (Charles River Laboratories, Wilmington, MA, USA, Catalog 027), female F1(C57BL/6xDBA/2) (Jackson Laboratories, Bar Harbor, ME, USA, catalog 100006), FcγR2b^-/- ^(Jackson, Catalog 002848), and TLR4^-/- ^mice (Stefanie Vogel, University of Maryland) were used [11]. All experiments, except those contracted to outside vendors, were approved by the Animal Care and Use Committee of the University of Maryland School of Medicine. Two contract research organizations paid by Gliknik, Inc., performed CIA experiments in separate mouse lines. Washington Biotechnology (Baltimore, MD, USA) used DBA1/J mice for their experiments, and Bolder-BioPATH (Boulder, CO, USA) used DBA/1OlaHsd mice (Harlan Laboratories, Frederick, MD, USA).

### Generation of stradomer and IgG2a Fc cDNA constructs

The Fc regions of the immunoglobulin sequence were fused to MDs either by splicing by overlap extension PCR or by using existing compatible restriction sites [12]. Polymerase chain reaction (PCR) primers were generated encoding the MD and an overlapping region of either the C or the N-terminus of the IgG Fc region and used for fusing the two sequences by PCR. The PCR products were then cloned into pcDNA3.3 by TA cloning (Invitrogen, Carlsbad, CA, USA) and confirmed by sequencing. Stradomers were transiently expressed in HEK293 cells after transfection.

### Purification of stradomers

Purification was done by using automated chromatography (Aktaxpress, GE, Piscataway, NJ, USA) and Hitrap Mabselect, protein A-derived ligand columns (GE 28-4082-56), followed by desalting on a HiPrep 26/10 Column (GE 17-5087-01). Stradomers were separated into homodimeric and multimeric fractions by using the standard Aktaxpress purification protocol and a GE Hiload 16/60 Superdex 200-pg size exclusion column (GE 17-1069-01).

### SDS-PAGE analysis

From 0.5 to 2 μg of nonreduced or reduced sample was loaded onto Nupage Novex 4-12% Bis-Tris Mini Gels (Invitrogen, Carlsbad, CA, USA, NPO322BOX) and were run according to the manufacturer's protocol (Invitrogen, IM-8042 Version H). Gels were washed with deionized water, stained for 30 minutes at room temperature with Bio-safe Coomassie Dye (Bio-Rad, Hercules, CA, USA, 161-0786), and destained with water.

### Surface plasmon resonance

Surface Plasmon resonance (SPR) was performed on a GE Healthcare Biacore 3000 instrument or on a Biacore T100. Recombinant mouse FcγRI, FcγRIIb, FcγRIII, FcγRIV, and SIGN-R1 (RnD Systems; Minneapolis, MN, USA) were immobilized onto a CM4 (FcγRs) or CM5 (SIGN-R1) chip by amine coupling. Samples were serially diluted in HBS-EP (running buffer) from 1,000 n*M *to 0.5 n*M *and injected at 20 μl/min, followed by a dissociation phase. Flow cells were regenerated with 1 *M *MgCl at 100 μl/min and then washed with HBS-EP (FcγRI required 10 m*M *glycine pH 2.0 to regenerate). A blank flow cell was used as a reference. All samples were run in duplicate or triplicate. The KD was calculated by using a 1:1 Langmuir kinetic model. Unless otherwise specified, all reagents were from GE Healthcare (Piscataway, NJ, USA). Because many of the preparations used in our studies contained multimers of diverse molecular masses, we standardized our analysis by assigning a molecular mass of 50 kDa to homodimer isolates and 150 kDa to all other protein preparations.

### Biolayer interferometry

Biolayer interferometry assay was performed on an Octet 96Red Instrument (ForteBio, Menlo Park, CA, USA) according to the manufacturer's instructions (Data Acquisition User Guide 6.4, ForteBio). For binding analysis, His-tagged mouse FcγRIIb (R&D Systems, Minneapolis, MN, USA, 1460-CD) and FcγRIII (R&D Systems 1960) were loaded on anti-penta-His Biosensors (ForteBio 18-5077) at 10 μg/ml in 1× kinetic analysis buffer (ForteBio,18-5032). After sensor-tip loading, protein association was measured by transfer of tips to preparations of either the monomeric (concentration 2,000 n*M *to 62.5 n*M*) or multimeric fractions (concentration, 100 n*M *to 3.13 n*M*) in 1× kinetics analysis buffer, and dissociation was measured by transfer of sensor tips to 1× kinetics buffer. Analysis was as described (Data Analysis User Guide 6.4ForteBio). Analysis was standardized, as described earlier by assigning a MW of 50 to homodimers and 150 to all other protein preparations.

### Collagen-induced arthritis model

Collagen-induced arthritis experiments were performed by two contract research organizations (CROs; Washington Biotechnology, Inc., Columbia, MD, USA; Bolder BioPATH Inc., Boulder, CO, USA). All arthritis experiments, except for the dose-response data for 2A-2H_C_, were generated by Washington Biotechnology by using the following protocol: On day 0, DBA1/J mice were immunized with 50 μl of a 4-mg/ml solution of Type II bovine collagen (Chondrex, Inc., Redmond, WA, USA, Cat. 20021) emulsified with an equal volume of complete Freund's Adjuvant (Sigma, St. Louis, MO, USA, Cat. 5506). A second vaccination was given on day 21 by using the same dose of Type II bovine collagen emulsified with an equal volume of incomplete Freund's Adjuvant. Mice were scored daily for signs of arthritis. Each paw was scored, and the sum of all four scores was recorded as the Arthritic Index (AI). The maximum possible AI was 16, as follows: 0, no visible effects of arthritis; 1, edema and/or erythema of one digit; 2, edema and/or erythema of two joints; 3, edema and/or erythema of more than two joints; and 4, severe arthritis of the entire paw and digits, including limb deformation and ankylosis of the joint. Starting on day 28 (treatment day 0), 10 collagen-immunized mice (all manifesting disease) were sorted into each of the treatment groups based on average AI (3.3). AI was measured for 14 treatment days, after which mice were euthanized. For the positive control group, mice were dosed orally with 10 mg/kg prednisolone daily. For the 2A-2H_C _group, mice were dosed every fourth day (day 0, day 4, day 8, and day 12) with 400 μg 2A-2H_C _(17.4 mg/kg). Fractionated 2A-2H_C _was given at a lower dose (250 μg, 10.9 mg/kg).

A second contract research organization, Bolder BioPATH Inc. (Boulder, CO, USA), independently generated dose-response data to 2A-2H_C _in a CIA model by using a slightly different protocol. In brief, male DBA/1OlaHsd (Harlan Laboratories, Frederick, MD, USA) mice were anesthetized with isoflurane and injected with 150 μl of Bovine Type II collagen (Elastin Products, Owensville, MO, USA), in Freund's complete adjuvant on day 0 and day 21 (Freund's complete adjuvant [with supplemental *Mycobacterium. tuberculosis*, 4 mg/ml], Difco). On study days 24 through 27, onset of arthritis occurred, and mice were randomized to treatment groups. Animals were randomized to a treatment group only after swelling was obviously established in at least one of four paws (score of 1 for the paw, minimum mean score of 0.25 for the animal). Attempts were made to assure approximately equal mean scores across all groups at the time of enrollment. Treatment was initiated after enrollment (treatment day 0). Mice were treated with intravenous 2A-2H_C _given at varying doses (10 mg/kg, 20 mg/kg, and 40 mg/kg). The first dose was administered on day 0 and then every fourth day. IVIG was given at a dose of 2 g/kg by intraperitoneal injection on day 0 and every fourth day. Dexamethasone was given orally at a dose of 0.2 mg/kg daily beginning on day 0. During this time, clinical scores were given for each of the paws (right front, left front, right rear, left rear) as follows: 0, normal; 1, 1 hind- or forepaw joint affected or minimal diffuse erythema and swelling; 2, 2 hind- or forepaw joints affected or mild diffuse erythema and swelling; 3, 3 hind- or forepaw joints affected or moderate diffuse erythema and swelling; 4, marked diffuse erythema and swelling, or four digit joints affected; and 5, severe diffuse erythema and severe swelling of the entire paw, unable to flex digits, or ankylosis, if present. Under this protocol, the mean score of all four paw scores, rather than a sum, was used for the scoring index.

### Induction and assessment of ITP

Platelet counts were measured by serial tail-vein nicking on days 1 through 5. To measure platelet counts, 10 μl of blood was diluted in 15 μl citrate buffer (0.105 *M*,///3.2%). The samples were analyzed for absolute platelet count on a Drew Scientific HemaVet 950FS hemocytometer that was standardized daily. Control-group mice received no pretreatment and no platelet depletion. ITP mice received only platelet depletion with MWReg30 α-platelet antibody (BD Biosciences, San Jose, CA, USA). Mice in the experimental groups received pretreatment with stradomers, murine IgG2a Fc, or IVIG on day 1, administered 16 to 24 hours before platelet depletion with MWReg30 each evening on days 2 through 4 (2 μg diluted in 200 μl PBS, given intraperitoneally). Mice pretreated with murine IgG2a Fc or stradomers received only a single dose (400 μg intravenous) on day 1. Mice pretreated with IVIG received daily doses (2 g/kg by intraperitoneal injection every morning on days 1 through 5).

In some experiments, administration of 2A-2H_C _on day 1 caused an initial decrease in platelet count before administration of MWReg30. Therefore, percentage platelet decrease is shown relative to the baseline measurement on day 2 by using the formula: % = measured platelet count/baseline platelet count on day 2. Experiments were done in replicate, as indicated, and all mice were included in pooled statistical analysis except for the following: for experiments testing the efficacy of the homodimeric versus multimeric fractions of 2A-2H_C _in preventing ITP, three replicate experiments were done with six mice per group. One mouse in the ITP group was excluded because of spurious platelet values. Two of 18 mice in the 2A-2H_C _group were excluded from day 5 analysis because of equipment failure that prevented measurement of platelet count.

### GVHD model

The GVHD model was previously described [13]. On day 0, BDF1 mice were given 4 × 10^7 ^C57BL/6 splenocytes and lymphocytes by tail-vein injection. Mice received no treatment, IgG2a Fc, or 2A-2H_C _(both given at 400 μg IV) on day 0 and day 4. In the preventive study, 2A-2H_C _was administered before adoptive transfer of B6 splenocytes and lymphocytes. On day 9, donor anti-host CTL activity was tested by ^51^Cr-release assay against H2d-expressing P815 cells (positive target) and EL4s that do not express H2d (negative control).

#### Injection of opsonized red blood cells (oRBCs)

One day before injecting oRBCs, mice were given either 40 mg of IVIG by intraperitoneal injection, 400 μg of murine IgG2a Fc by intravenous injection, or 400 μg of 2A-2H_C _by intravenous injection. Fluorescence-labeled oRBCs were generated as described by Song *et al*. (2003) [14]. Erythrocytes were then washed 3 times with PBS and resuspended in 5 ml of PBS. Mice were given tail-vein injections of 200 μl of oRBCs. Mice were then bled by tail-vein nicking at 3, 30, 60, 90, and 165 minutes after injection. Blood (10 μl) was collected into a tube containing an equal volume (10 μl) of 1% EDTA in PBS. After mixing, 1 μl of the sample was diluted in 300 μl of FACS buffer and used for flow cytometry. The percentage of PKH26-labeled oRBCs in circulation was measured with flow cytometry. The percentage of fluorescent erythrocytes at the 3-minute time point was considered the baseline (100%).

### Statistics

Statisticians had access to all primary data. For the platelet data, Wilcoxon rank-sum tests were used to compare percentages between groups. For the Arthritis Index data, linear mixed-effects models with either one or two slopes were used to compare the Arthritis Index over time between groups. For the platelet and the RBC data, percentages of baseline values were calculated for each post-baseline time point and presented in the plots as means ± SEM. Wilcoxon rank-sum tests were used to compare percentages between groups at some later time points at which we expected differences to have developed between groups (days 4 and 5 for platelet data, and 60 minutes, 90 minutes, and 165 minutes after injection for RBC data). Unless otherwise indicated, all comparisons reported were made against the diseased with no-treatment group (ITP, CIA, or injected oRBCs with no pretreatment). Bonferroni's correction was used for significance where multiple comparisons were made, with the intended number of comparisons reported.

## Results

### Stradomers exist as highly ordered multimers that can be fractionated by size

**Figure 1:**
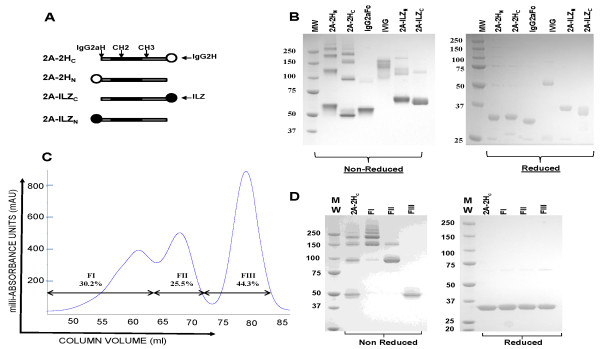
**Stradomers form highly ordered multimers, which can be fractionated by size**. **(A) **Stradomers consist of the murine IgG2a hinge, CH2, and CH3 domains (abbreviated as 2A), linked to a multimerization domain (MD) at the carboxy (C) or amino (N) terminus. The MD is either the human IgG2 hinge (2H) or an isoleucine zipper (ILZ). **(B) **SDS-PAGE of the stradomer proteins. Homodimeric 2A-2H_C _bands have a molecular mass of 55 kDa. Multimeric bands have a mass of 150 kDa or more. **(C) **The 2A-2H_C _protein was fractionated on a GE Highload Phenyl Sepharose High-Performance column (GE 17-1066-01) by using a standard protocol. The sample was run at a rate of 3 ml/min, and 2-ml fractions were collected. UV spectrometry at 280 nm was used to quantify protein in each fraction, yielding a histogram readout characterized by peaks representing the different fractions. **(D) **SDS-PAGE of unfractionated 2A-2H_C_, fraction I, fraction II, and fraction III. Homodimeric 2A-2H_C _bands have a mass of 55 kDa. Multimeric bands are higher at ≥ 100 kDa.

To evaluate the potential of the IgG2 hinge as an MD, we created cDNA constructs in which the hinge region of human IgG2 was linked to either the carboxy (2A-2H_C_) or amino (2A-2H_N_) termini of murine IgG2a Fc. In parallel, we developed compounds with the previously described ILZ at the carboxy (2A-ILZ_C_) or amino termini (2A-ILZ_N_) (Figure 1A) [15]. None of these fusion proteins contains an Fab region. We named the resultant proteins "stradomers" to distinguish them from naturally occurring homodimeric and multimeric immunoglobulins.

On purification, analysis of these compounds with SDS-PAGE revealed that both of the stradomers containing the isolated IgG2 hinge region exist as homodimers (50 kDa) and as highly ordered multimers, which occur at a far higher percentage than found with recombinant IgG2a Fc (Figure 1B). The fact that these multimers are present under denaturing, but not reducing conditions, suggests that they are covalently linked. Although both ILZ compounds were also composed of homodimers and multimers, the presence of higher-order multimers was less than that observed for the 2H stradomers. This lack of higher-order multimers was most pronounced for the 2A-ILZ_C _protein. As expected, recombinant IgG2a Fc was present at the appropriate size for a homodimer (45 kDa). In addition, a less-intense band, which likely represents a multimer, was present at approximately 90 kDa (Figure 1B).

As a first step toward characterizing the function of these stradomers, we isolated the homodimeric and multimeric forms of one representative stradomer, 2A-2H_C_. SDS-PAGE analysis of the resultant fractions revealed that the higher-order multimers could be separated from the homodimeric fractions with a relatively high degree of purity (Figure 1C, D). These data demonstrate that incorporation of a novel human IgG2H MD onto either the amino or carboxy terminus of mouse IgG2a Fc results in a fusion protein, which forms highly ordered Fc multimers that can be fractionated according to Fc valency.

### Stradomer binding to low-/intermediate-affinity canonic FcγRs and SIGN-R1 directly correlates with the degree of multimerization

Low-/intermediate-affinity FcγRs, including FcγRIIb, FcγRIII, and FcγRIV, are recognized to form more-stable associations with Fc-bearing immune complexes than with nonmultimerized Fc of the appropriate isotype [16]. Furthermore, the mechanisms by which both IVIG and Fc-bearing immune complexes mediate tolerance are reported to depend on engagement of selected low/intermediate receptors [2]. Based on these data, we sought to determine whether these fully recombinant stradomers could effectively engage the canonic FcγRs and to correlate the stability of these interactions with Fc valency.

**Figure 2:**
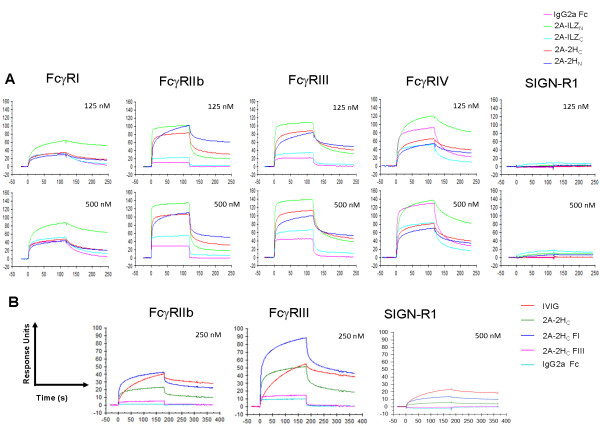
**The ability of stradomers to engage canonic FcγRs and SIGN-R1 correlates with their degree of multimerization**. Recombinant mouse FcγRI, FcγRIIb, FcγRIII, FcγRIV, and SIGN-R1 were immobilized onto a CM4 (FcγRs) or CM5 (SIGN-R1) chip by amine coupling. The stradomers and Fc-modified antibodies were injected over the immobilized receptors. The SPR sensorgrams depict the association and dissociation phases of the injected proteins with the receptors. Increased affinity is indicated by higher relative RU. **(A) **SPR sensorgrams of stradomers binding to the canonic FcγRs and the C-type lectin SIGN-R1 are shown. **(B) **The 2A-2H_C _stradomer was separated into multimer-enriched (I) and homodimer-enriched (III) fractions. SPR sensorgrams indicate binding of IgG2a Fc, IVIG, whole 2A-2H_C_, fraction I, and fraction III to FcγRIIb, FcγRIII, and SIGN-R1.

Evaluation of stradomer interactions with murine FcγRs by surface plasmon resonance (SPR) revealed that, in comparison to the transient associations observed with recombinant IgG2a Fc, the 2A-2H_C_, 2A-2H_N_, and 2A-ILZ_N _proteins formed stable interactions with FcγRI, FcγRIIb, FcγRIII, and FcγRIV. The interactions of 2A-ILZ_C _with these receptors were similar to those of IgG2a Fc, consistent with the lack of highly ordered multimer formation. Additionally, based on reports that suggest that the C-type lectin, SIGN-R1, regulates the antiinflammatory effects of IVIG through specific interactions with α-2,6-sialylated Fcs [17], we also evaluated the ability of the stradomers to interact with SIGN-R1. The ability of unfractioned stradomers to bind SIGN-R1 was superior to that of IgG2a Fc but more limited in comparison with the canonic receptors, and the stability of these associations was generally less than that observed for interactions with the canonic FcγRs (Figure 2A, Table 1).

**Table 1:** Binding analysis of 2H- and ILZ-based stradomers to individual FcRs

	FcγRI	FcγRIIb	FcγRIII	FcγRIV	SIGN-R1
IgG2a Fc	17.4 (4.07)	289 (1.48)	248 (2.9)	27.8 (25.4)	n.s.^a^
2A-ILZ_N_	6.71 (52.9)	3.8 (51.3)	2.3 (85.4)	.46 (88.3)	134 (0.43)
2A-ILZ_C_	63.4 (7.62)	455 (11)	151 (16.1)	63.6 (20)	43.9 (0.77)
2A-2H_N_	16.3 (7.62)	3.4 (12.1)	4.4 (24.9)	7.6 (11.3)	109 (0.11)
2A-2H_C_	9.12 (9.97)	3.3 (29.2)	4.2 (40.6)	3.03 (18.9)	n.s.^a^

KD (left, in n*M*), and χ^2 ^(right, in parenthesis). ^a^Insufficient binding to calculate KD.

To place our findings in context with previous reports demonstrating that the valency of Fc-containing compounds affects interactions with their cognate receptors [16], we evaluated binding of the individual fractions of 2A-2H_C _with FcγRIIb, FcγRIII, and SIGN-R1 by SPR (Figure 2B), and with FcγRIIb and FcγRIII by Biolayer Interferometry (see Additional File 1, Figure S1). Consistent with the findings of others, higher-order multimers of 2A-2H_C _formed more-stable interactions with both FcγRIIb and FcγRIII than did the smaller isolates. Unexpectedly, the higher-order isolates also bound with greater avidity to SIGN-R1, suggesting that this may be a low- to moderate-affinity receptor. Taken in concert, these data demonstrate that fully recombinant stradomers can recapitulate the binding avidity of Fc-bearing immune complexes.

### Stradomers can effectively treat collagen-induced arthritis

**Figure 3:**
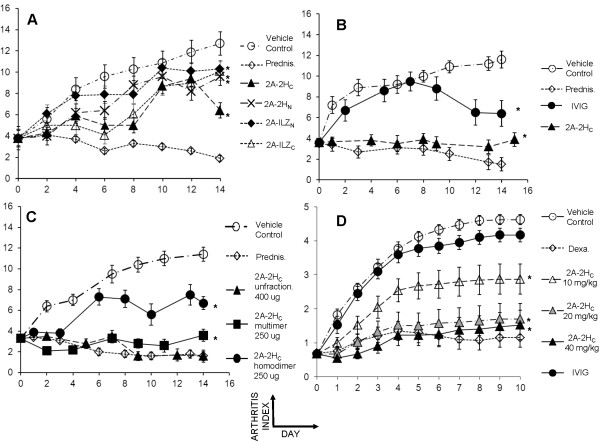
**Stradomers effectively treat CIA**. Stradomers were tested in a treatment model of murine CIA. Treatment was initiated after mice had an average arthritis score of 3.3. Each paw was scored individually for arthritis (0 to 4), and the sum of all four paws was used as the score for the animal (maximum of 16). The positive treatment control for these experiments was oral prednisolone at 10 mg/kg daily. **(A) **Direct comparison of stradomers in the CIA model. **(B) **Comparison of 2A-2H_C _efficacy against IVIG. **(C) **Comparison of the 2A-2H_C _homodimeric and multimeric fractions. (*Significant compared with no treatment, with Bonferroni correction). The 2A-2H_C _dose-response experiments were performed by a separate contract organization that used a different arthritis-scoring system. Each paw was scored on a scale of 0 to 5. The average of all four paws (rather than the sum) was used as the score for the animal. **(D) **Dose-response curve of 2A-2H_C_. Treatment control for this experiment was oral dexamethasone at 0.2 mg/kg daily.

To begin to understand the function of the stradomers *in vivo*, we used a CIA model thought to recapitulate closely many of the essential features of rheumatoid arthritis. CIA studies were performed at two different contract research organizations, with selected experiments blinded to the test articles. Animals receiving the drug experienced only limited toxicities, the major manifestations of which included transient piloerection and a brief period of lethargy. In the CIA model, all stradomers showed pronounced therapeutic effects in treating CIA (*P *< 0.0001 for 2A-2H_C_, 2A-2H_N_, and 2A-ILZ_C_; *P *= 0.0021 for 2A-ILZ_N_) (Figure 3A). Interestingly, the 2A-ILZ_C _fusion protein, which forms limited higher-order multimers in solution, was able to alleviate CIA significantly (*P *< 0.0001). Although IVIG was also therapeutic, the kinetics and potency of this effect were delayed and less robust than those observed with 2A-2H_C _(*P *< 0.0001 for IVIG) (Figure 3B).

Based on the finding that 2A-ILZ_C_, which had a low percentage of multimers, can alleviate CIA, we isolated the homodimeric and multimeric fractions of 2A-2H_C _to determine the importance of Fc multimers in this model. Whereas the homodimeric fraction of 2A-2H_C _demonstrated some therapeutic benefit in alleviating CIA, this treatment effect was less robust than that observed with the multimeric fraction (*P *< 0.0001 for homodimeric and multimeric fractions of 2A-2H_C_) (Figure 3C). These findings are consistent with the idea that Fc homodimers may have some therapeutic benefit in selected models based on their ability to block transiently FcγRs and to decrease the half-life of pathogenic autoantibodies by competing for binding with the neonatal receptor (FcRn) [18]. It is also the case that murine IgG2a Fc, unlike human IgG1 Fc, autodimerizes (Figure 1B), which may contribute to the activity of IgG2a Fc "homodimers" in murine models.

Finally, to understand whether our findings were dose dependent, we had a third-party CRO blinded to dose and control evaluate doses of 10, 20, and 40 mg/kg in the CIA model. Our data indicate that a dose-dependent difference exists in potency (Figure 3D). These findings suggest that although a dose-response exists to the stradomers in CIA, a therapeutic threshold may exist.

### Stradomers can effectively prevent idiopathic thrombocytopenic purpura

**Figure 4:**
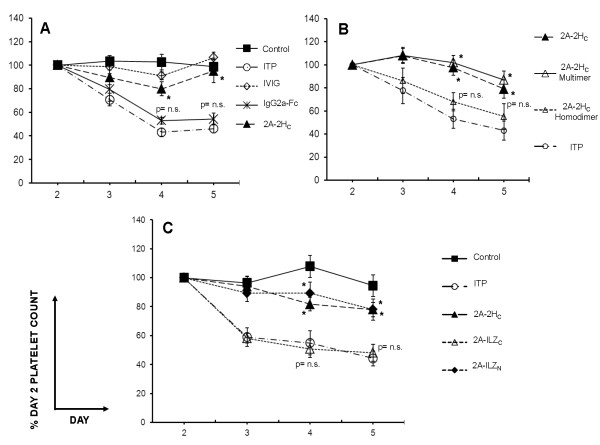
**Stradomers effectively prevent ITP**. **(A) **Mice were pretreated with IgG2a Fc, 2A-2H_C_, or IVIG on day 1 before platelet depletion with MWReg30. Day 2 platelet counts before MWReg30 administration were considered baseline (100%) (*Significant with Bonferroni correction for the four comparisons indicated; *n *= 11 to 12 per group pooled from two separate experiments). **(B) **2A-2H_C _was fractionated into homodimer-enriched (fraction III) and multimer-enriched (fraction I) fractions and tested in the ITP model (*significant with Bonferroni correction for the six comparisons indicated; *n *= 12 to 18 per group pooled from three experiments, two testing unfractionated 2A-2H_C_, fraction I, and fraction III, and one testing only unfractionated 2A-2Hc and fraction I). **(C) **The different stradomers were directly compared in the ITP model. (*n *= 11 to 12 mice per group, pooled from two separate experiments. *Significant with Bonferroni correction).

To extend our findings, we evaluated the therapeutic utility of stradomers in murine models of ITP and graft-versus-host disease (GVHD). The ITP model differs from CIA in that anti-platelet antibody is directly administered to the host, and thus is not dependent on secondary antibody development as a result of antigen priming/boosting. In the ITP model, a single intravenous dose of 400 μg of 2A-2H_C _protected mice against platelet loss (*P *< 0.0001, days 4, 5), whereas recombinant murine IgG2a Fc was less effective (*P *= 0.0145; *P *= 0.178 days 4, 5, neither significant with the Bonferroni correction) (Figure 4A). Fractionation of 2A-2H_C _revealed that the observed protective effects were primarily attributable to the multimeric (*P *< 0.0001, days 4, 5) rather than the homodimeric fraction (*P *= 0.0093; *P *= 0.140 days 4, 5, neither significant with the Bonferroni correction) (Figure 4B). Consistent with previous mouse ITP prevention studies, daily high-dose administration of IVIG provided nearly complete protection against platelet loss. Functionally, although 2A-ILZ_N _conferred significant protection from MWReg30-induced platelet depletion (*P *= 0.0056; *P *= 0.0001; days 4, 5), unlike that in the CIA model, the 2A-ILZ_C _protein at the same dose was not effective (*P *= 0.80; *P *= 0.76 days 4, 5) (Figure 4C).

In some, but not all, individual experiments, we observed a significant platelet decrease 16 to 24 hours after infusion of 2A-2H_C_, before the administration of MWReg30 (see Additional File 2, Figure S2A). Detailed studies in TLR-4^-/- ^mice, which lack the endotoxin receptor [11], revealed that this effect is not due to contaminating endotoxin. However, flow cytometry-based platelet-binding studies suggest that these observations may be secondary to the ability of 2A-2H_C _to bind to platelets, resulting in their transient sequestration (see Additional File 2, Figure S2B). Taken in concert, our findings confirm that the protection conferred by recombinant IgG2a Fc fusion compounds against the development of ITP is not restricted to an individual MD or MD location, and suggest that protection may be related to the percentage and/or size of multimers in the preparation, as is the case with IVIG aggregates [4,16].

Importantly, unlike ITP and CIA, in which antibody responses are recognized to play an important role in disease pathogenesis, 2A-2H_C _was not effective in either the prevention or the treatment of GVHD (see Additional File 3, Figure S3). These differences in therapeutic utility between model systems might be explained by the fact that this GVHD model is primarily T-cell mediated, a fact that may be exploited in future studies as a means to determine the mechanisms by which stradomers mediate their biologic effects [13].

### 2A2H_C _inhibits depletion of circulating opsonized RBCs

Our data indicate that stradomers alleviate CIA and prevent ITP, suggesting that they may represent an advance toward a recombinant analogue of IVIG. The primary hurdle to creating a fully recombinant IVIG is the absence of a clear mechanism of action. Specifically, IVIG is postulated to mediate its antiinflammatory effects through multiple mechanisms, including, but not limited to, (a) saturating the neonatal receptor, FcRn, so that pathogenic autoantibodies are more rapidly cleared from the circulation; (b) binding to SIGN-R1/DC-SIGN and/or the activating FcγIIIa receptor, with subsequent upregulation of the FcγRIIb inhibitory receptor; and (c) competitive inhibition of Fcγ receptor signaling [19-21]. Further complicating matters are the facts that these mechanisms are not mutually exclusive and that the therapeutic effects of IVIG may prove to be disease specific.

**Figure 5:**
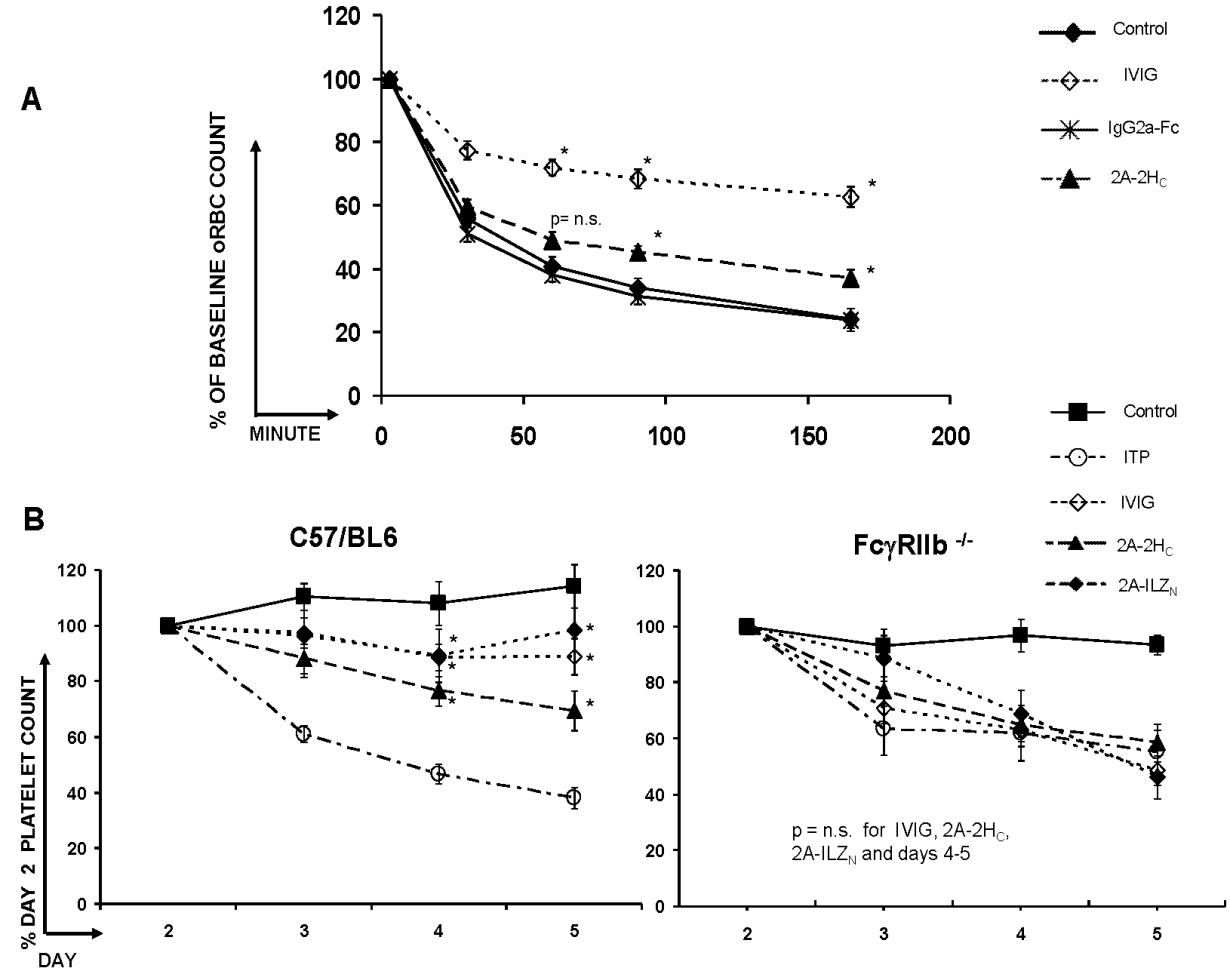
**The RES and FcγRIIb are relevant to stradomer-mediated protection against ITP**. **(A) **Mice were pretreated with 2A-2Hc or IgG2a Fc for 1 day before injection of fluorescently labeled, opsonized red blood cells (oRBCs) to test whether pretreatment protects against clearance of circulating oRBCs by the RES. Data shown as percentage-labeled oRBCs/percentage-labeled oRBCs at 3 minutes × 100 (*Significant with Bonferroni adjustment for the six comparisons indicated; *n *= 12 mice/group pooled from two separate experiments including these specific treatment groups). **(B) **To determine whether the stradomer-mediated protection against ITP in mice is dependent on FcγRIIb, 2A-2H_C _and 2A-ILZ_N _were tested simultaneously in C57/Bl6 mice with functional FcγRIIb (left) and in FcγRIIb^-/- ^mice (right) (*Significant with Bonferroni adjustment for the six comparisons indicated. *N *= 17 to 18 mice per group, except 2A-ILZ_N _(*n *= 6), pooled from three separate experiments, one of which included the 2A-ILZ_N _stradomer).

It has been postulated that IVIG-mediated protection against ITP is partly due to its capacity to saturate the reticuloendothelial system (RES), preventing phagocytosis of autoantibody opsonized platelets. To evaluate this possibility further, we tested the ability of 2A-2H_C _to block the RES, by using an opsonized RBC (oRBC) depletion model [22]. Our findings reveal that, like IVIG, 2A-2Hc inhibits the removal of oRBCs from the circulation, indicating that 2A-2H_C _likely interferes with the RES function (Figure 5A). Furthermore, because these RBCs were opsonized *ex vivo*, our data suggest that the function of 2A-2H_C _in ITP cannot solely be attributed to blockade of the FcRn, which has the potential to decrease the half-life of the MWReg30 anti-platelet antibody used in our ITP model [23].

### 2A-2H_C _protection requires FcγRIIb

Next, we sought to define the role of the inhibitory FcγRIIb receptor in 2A-2H_C_-mediated protection against ITP. The decision to interrogate FcγRIIb was based on previous studies that have clearly documented that this receptor is necessary for the protective effects of IVIG in ITP models [24,25]. By using the same strain of FcγRIIb^-/- ^mice studied by Siragam and colleagues [25], we observed a baseline increase in the hematocrit and a decrease in the platelet counts of FcγRIIb^-/- ^mice, which limits the dynamic range of the ITP assay (see Additional File 4, Figure S4). Consistent with the findings of other investigators, our data convincingly demonstrate that FcγRIIb is required for the protective effects of IVIG in a model of ITP (Figure 5B) [24,25]. Our data also suggest that 2A-2H_C_- and 2A-ILZ_N_-mediated protection against ITP is likely dependent on FcγRIIb binding, as neither agent conferred statistically significant protection against platelet loss in FcγRIIb^-/- ^mice (Figure 5B). Importantly, the reduced range of this assay introduces a potential source of bias, which mandates caution when interpreting these data.

## Discussion

Our data demonstrate that we have created recombinant stradomers that are capable of treating a murine model of arthritis and preventing the development of ITP. Although other investigators have shown the utility of Fc-bearing immune complexes in murine models of autoimmunity [1,2,26,27], to the best of our knowledge, this is the first report to describe the generation and production of fully recombinant Fc multimers. The recombinant nature of these fusion proteins will likely facilitate clinical translation with Fc isotype-appropriate human analogues. Furthermore, we show for the first time that the IgG2H can be used as an MD. Based on its limited size, and its existence as a normal component of human IgG2, this peptide may have broad utility for protein multimerization. Finally, because stradomers can be generated on species-specific Fc backbones and can be readily fractionated by size, they may have value both in defining how Fc:FcγR interactions modulate inflammation and in acting as controls for antibody-based studies *in vivo*.

Whereas other investigators have laid the groundwork for the use of Fc-bearing aggregates in the treatment of autoimmune diseases [2], to the best of our knowledge, no one has generated fully recombinant Fc multimers with therapeutic intent. To accomplish this task, we sought to identify low-molecular-weight peptides that could induce Fc multimerization. Human IgG2 exists as a small percentage of dimers (dimers of the natural homodimer) in sera, and this dimerization is dependent on the hinge region [8-10]. Based on this knowledge, we postulated that by engineering this human IgG2H sequence onto the amino or carboxy terminus of mouse IgG2a, we could generate fully recombinant Fc multimers.

SDS-PAGE analysis revealed that the resultant 2A-2H proteins exist as highly ordered multimer bands that can be fractionated by size. In comparison with the ILZ-based stradomers, the degree of multimerization was higher for the IgG2H-bearing fusion proteins. Based on previous work demonstrating the import of the low-affinity canonic FcγRs and SIGN-R1 in modulating the antiinflammatory and antiautoimmune effects of IVIG and/or immune complexes comprising Fc regions, we initially sought to understand how stradomers interacted with these receptors [2,17,24,25,28,29]. Selected stradomers demonstrate strong interactions with the low-/intermediate-affinity FcγRs, the stability of which are directly related to their degree of multimerization. Surprisingly, the multimeric fraction of 2A-2H_N _displayed a higher affinity for SIGN-R1 than its homodimeric analogue, suggesting that SIGN-R1 may be a low-affinity receptor for IgG2a.

To evaluate the function of these stradomers *in vivo*, we compared their efficacy with "gold standard" therapies (that is, prednisolone, dexamethasone, and IVIG), in three unique models of autoimmunity. The stradomers evaluated herein contained a murine IgG2a Fc backbone without any Fab fragment, limiting the potential for interspecies cross-reactivity to either the human IgG2 hinge sequence and/or conformational Fc changes induced by this peptide. Stradomer administration was associated with minimal toxicity, the primary manifestations of which were transient piloerection and a brief period of lethargy. In the CIA model, stradomers demonstrated therapeutic efficacy in a dose-dependent fashion. Interestingly, although IVIG partially ameliorated CIA, the kinetics and potency of this response differed from that observed with 2A-2H_C_. These findings are consistent with the knowledge that IVIG has variable therapeutic efficacy in rheumatoid arthritis [30] and provide a rationale for evaluating fully recombinant Fc multimers in this disease.

The fact that clinical benefit in both CIA and ITP was observed with stradomers composed of two unique MDs suggests that our findings are not specifically related to the multimerization peptide. Consistent with previous reports describing the mechanism of IVIG in ITP models, stradomer-mediated protection against platelet loss may in part be attributable to saturation of the RES, and it requires FcγRIIb [24,25,31]. Importantly, based on our finding that FcγRIIb^-/- ^mice have lower platelet counts at baseline than do wild-type C57/Bl6 mice, the dynamic range of the ITP assay is reduced, and these data must be interpreted with caution. Furthermore, unlike the ITP model, in which multimerization was required for protection against platelet loss, the homodimeric fraction of 2A-2H_C _demonstrated therapeutic efficacy in CIA. However, the potency of the homodimeric fraction was less than that observed for the isolated multimers. These data suggest that the mechanisms through which stradomers mediate their therapeutic effects could be distinct in these two model systems.

Based on their efficacy in animal models of CIA and ITP, the human analogues of these stradomers hold promise for the amelioration of selected human autoimmune diseases. In addition, we hypothesize that because of its small size and existence as a natural component of human IgG2, the human IgG2 hinge peptide will have broad utility as a protein MD with limited associated immunogenicity. Finally, although our study was not designed to provide detailed mechanistic insights into the means by which Fc multimers ameliorate autoimmunity, stradomers, and the isolated fractions thereof, have the potential to serve as a platform to define the mechanisms by which Fc:FcγR interactions regulate inflammation as species-specific controls for mAb studies *in vivo*.

## Conclusions

This study demonstrates that fully recombinant forms of murine IgG2a Fc containing unique multimerization domains, termed stradomers, have the capacity to alleviate CIA and to prevent ITP in mice. Stradomers form multimeric fractions in solution that are comparable to the naturally occurring aggregates found in IVIG. Furthermore, they demonstrate strong interactions with the low-/intermediate-affinity FcγRs, the stability of which are directly related to their degree of multimerization. Fully humanized versions of these unique proteins may have translational applications for treating arthritis and other selected autoimmune diseases.

## Abbreviations

AI: arthritic index; CIA: collagen-induced arthritis; CRO: contract research organization; FcγR: Fc gamma receptor; FcRn: neonatal receptor; GVHD: graft-versus-host disease; IgG2H: human IgG2 hinge sequence; ILZ: isoleucine zipper; ITP: idiopathic thrombocytopenic purpura; IVIG; intravenous immune globulin; MD: multimerization domain; SPR: surface plasmon resonance; 2A-2H_C_: stradomer with hinge of human IgG2 linked to the carboxy terminus of murine IgG2a Fc; 2A-2H_N_: stradomer with hinge of human IgG2 linked to the amino terminus of murine IgG2a Fc; 2A-ILZ_C_: stradomer with the isoleucine zipper linked to the carboxy terminus of murine IgG2a Fc; 2A-ILZ_N_: stradomer with the isoleucine zipper linked to the amino terminus of murine IgG2a Fc.

## Competing interests

Scott Strome is the cofounder and major stockholder of a biotechnology company, Gliknik Inc. Gliknik, Inc., is both a coinventor and a licensee of the stradomers. The studies performed in Dr. Strome's laboratory were financed by Gliknik, Inc., through a sponsored research agreement through the University of Maryland School of Medicine. The University is managing Dr. Strome's conflict of interest according to its established financial conflict-of-interest policies and procedures. Dr. Schulze is also a stockholder of Gliknik, Inc., and he has received partial salary support through Gliknik, Inc. The University is aware of his conflict. David Block, Henrik Olsen, and Emmanuel Merigeon are all employees of Gliknik, Inc. They are compensated by Gliknik, Inc., and hold equity interests in the company. Because Gliknik, Inc., is a co-inventor/licensee of the technology, the results could affect the value of the shares owned by Scott Strome, Dan Schulze, David Block, Henrik Olsen, and Emmanuel Merigeon. Gliknik, Inc. has applied for patents related to the compounds published in this report. Dr. Strome has received consulting fees from Gliknik, Inc., and they sponsor research in his laboratory through an SRA. Dr. Schulze has received partial salary support through Gliknik, Inc.

## Authors' contributions

AJ and SS designed the research, performed ITP and oRBC experiments, and analyzed the data. RV performed ITP and oRBC experiments. EB performed Biacore and oRBC experiments. YS and KT performed graft-versus-host experiments. HO, EYM, and DB manufactured stradomers, conducted SDS-PAGE experiments, and contracted CIA experiments to two independent contract research organizations. LC and CL performed statistical analysis. DS helped design the stradomers. All authors read the manuscript and approved it for publication.

## Supplementary Material

Additional file 1 **Figure S1, 2A-2H_C _multimer size correlates with binding stability to FcγRIIb and FcγRIII**. This figure shows Octet biosensor assay data demonstrating that stradobody multimerization results in more stable associations with FcγRIIb and FcγRIIIa. Click here for file

Additional file 2 **Figure S2, 2A-2H_C _can cause an initial decrease in platelet count before platelet depletion with MWReg30 that may be the result of platelet sequestration by 2A-2H_C_**. This figure demonstrates that 2A-2H_C_, in some experiments, caused an initial decrease in platelet count before administration of the platelet-depleting antibody MWReg30. Click here for file

Additional file 3 **Figure S3, 2A-2H_C _does not prevent graft-versus-host disease**. This figure demonstrates that 2A-2H_C _cannot prevent T-cell-mediated graft-versus-host disease in a murine model. Click here for file

Additional file 4 **Figure S4, The FcγRIIb^-/- ^and "wild type" C57/BL6 have differing baseline platelet and red blood cell counts**. This figure demonstrates that FcγRIIb^-/- ^and "wild type" C57/BL6 have differing baseline platelet counts, which could potentially affect the dynamic range of ITP assay in FcγRIIb^-/- ^mice. Click here for file
