# Supplementary material for: Fully recombinant IgG2a Fc multimers (stradomers) effectively treat collagen-induced arthritis and prevent idiopathic thrombocytopenic purpura in mice
Source: Arthritis Res Ther. 2012 Aug 20;14(4):R192. doi: 10.1186/ar4024 (PMC3580588; doi:10.1186/ar4024)
Supplement: Additional file 1 — Figure S1, 2A-2HC multimer size correlates with binding stability to FcγRIIb and FcγRIII. This figure shows Octet biosensor assay data demonstrating that stradobody multimerization results in more stable associations with FcγRIIb and FcγRIIIa. [file ar4024-S1.PPT]

## Slide 1
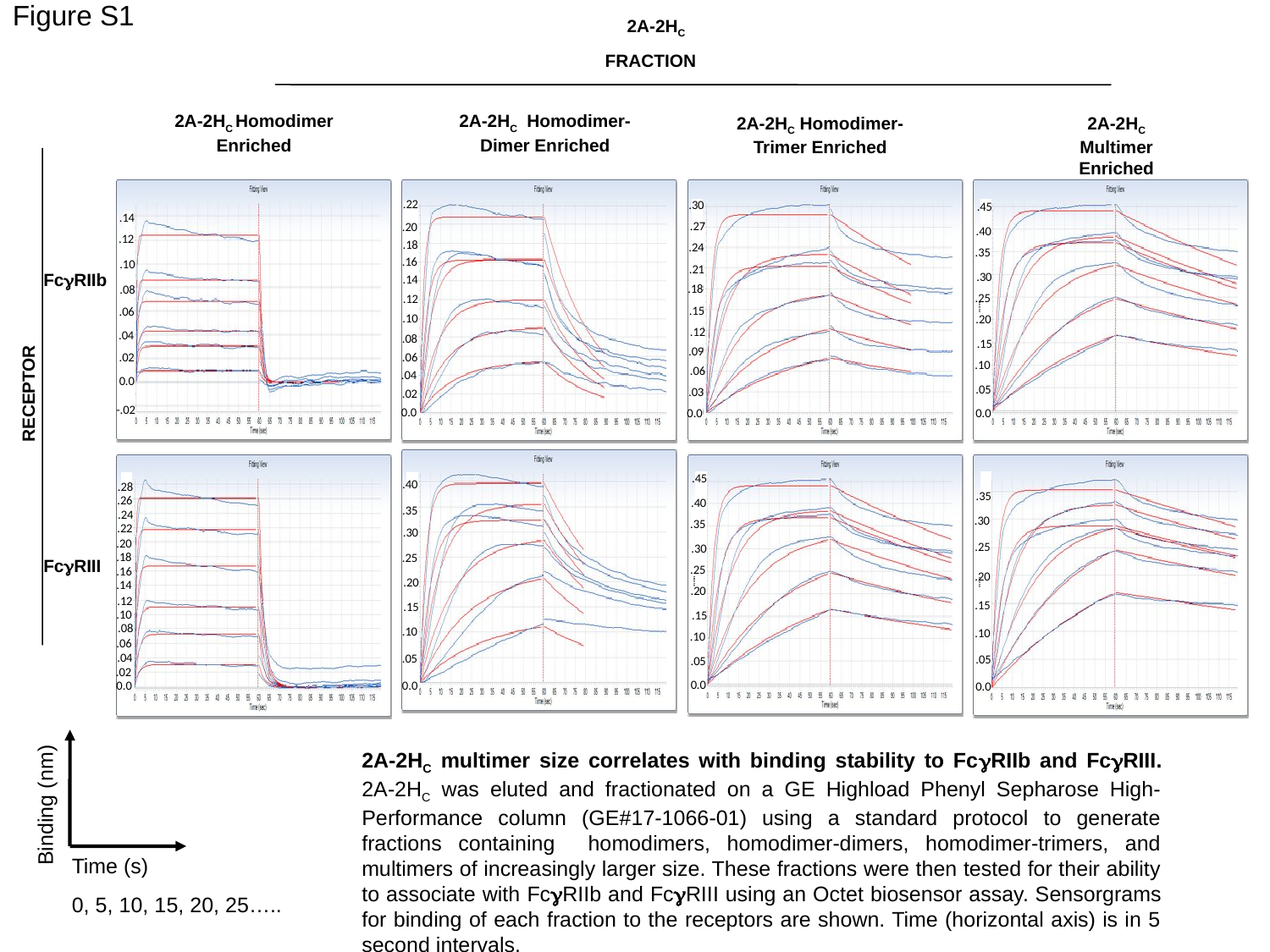

Figure S1
2A-2HC
FRACTION
2A-2HC Homodimer Enriched
2A-2HC Homodimer-Dimer Enriched
2A-2HC Homodimer-Trimer Enriched
2A-2HC Multimer Enriched
.22
.30
.45
.14
.27
.20
.40
.12
.18
.24
.35
.16
.10
.21
 FcγRIIb
.30
.14
.18
.08
.25
.12
.15
.06
.10
.20
RECEPTOR
.12
.04
.08
.15
.09
.02
.06
.10
.06
.04
0.0
.05
.03
.02
-.02
0.0
0.0
0.0
.45
.40
.28
.35
.26
.40
.35
.24
.30
.35
.22
.30
.20
.25
.30
.18
.25
 FcγRIII
.25
.16
.20
.20
.14
.20
.12
.15
.15
.10
.15
.08
.10
.10
.10
.06
.04
.05
.05
.05
.02
0.0
0.0
0.0
0.0
2A-2HC multimer size correlates with binding stability to FcγRIIb and FcγRIII. 2A-2HC was eluted and fractionated on a GE Highload Phenyl Sepharose High-Performance column (GE#17-1066-01) using a standard protocol to generate fractions containing homodimers, homodimer-dimers, homodimer-trimers, and multimers of increasingly larger size. These fractions were then tested for their ability to associate with FcγRIIb and FcγRIII using an Octet biosensor assay. Sensorgrams for binding of each fraction to the receptors are shown. Time (horizontal axis) is in 5 second intervals.
Binding (nm)
Time (s)
0, 5, 10, 15, 20, 25…..
