# Supplementary material for: Fully recombinant IgG2a Fc multimers (stradomers) effectively treat collagen-induced arthritis and prevent idiopathic thrombocytopenic purpura in mice
Source: Arthritis Res Ther. 2012 Aug 20;14(4):R192. doi: 10.1186/ar4024 (PMC3580588; doi:10.1186/ar4024)
Supplement: Additional file 2 — Figure S2, 2A-2HC can cause an initial decrease in platelet count before platelet depletion with MWReg30 that may be the result of platelet sequestration by 2A-2HC. This figure demonstrates that 2A-2HC, in some experiments, caused an initial decrease in platelet count before administration of the platelet-depleting antibody MWReg30. [file ar4024-S2.PPT]

## Slide 1
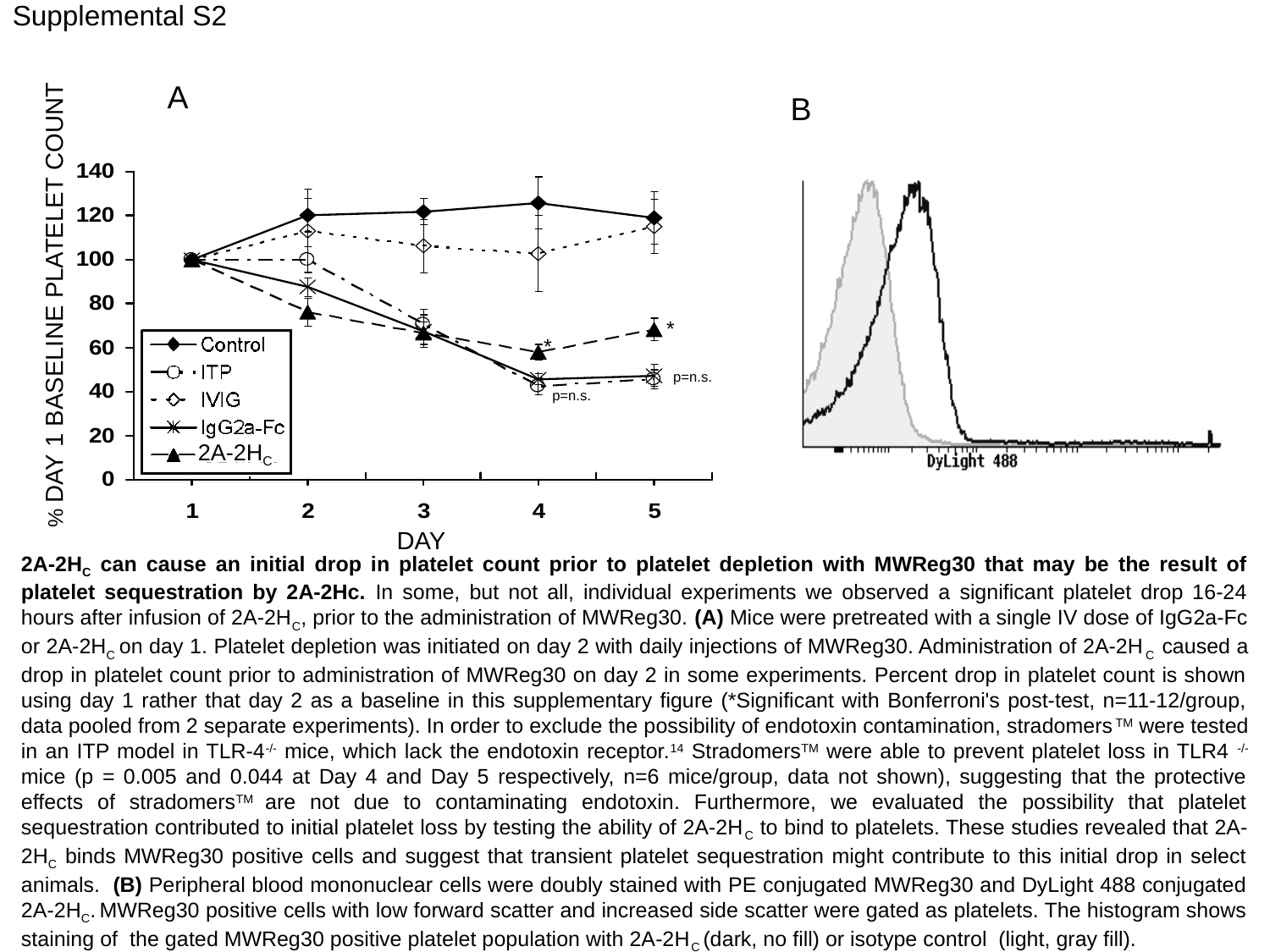

Supplemental S2
A
B
% DAY 1 BASELINE PLATELET COUNT
 *
 *
2A-2HC
p=n.s.
p=n.s.
 DAY
2A-2HC can cause an initial drop in platelet count prior to platelet depletion with MWReg30 that may be the result of platelet sequestration by 2A-2Hc. In some, but not all, individual experiments we observed a significant platelet drop 16-24 hours after infusion of 2A-2HC, prior to the administration of MWReg30. (A) Mice were pretreated with a single IV dose of IgG2a-Fc or 2A-2HC on day 1. Platelet depletion was initiated on day 2 with daily injections of MWReg30. Administration of 2A-2HC caused a drop in platelet count prior to administration of MWReg30 on day 2 in some experiments. Percent drop in platelet count is shown using day 1 rather that day 2 as a baseline in this supplementary figure (*Significant with Bonferroni's post-test, n=11-12/group, data pooled from 2 separate experiments). In order to exclude the possibility of endotoxin contamination, stradomersTM were tested in an ITP model in TLR-4-/- mice, which lack the endotoxin receptor.14 StradomersTM were able to prevent platelet loss in TLR4 -/- mice (p = 0.005 and 0.044 at Day 4 and Day 5 respectively, n=6 mice/group, data not shown), suggesting that the protective effects of stradomersTM are not due to contaminating endotoxin. Furthermore, we evaluated the possibility that platelet sequestration contributed to initial platelet loss by testing the ability of 2A-2HC to bind to platelets. These studies revealed that 2A-2HC binds MWReg30 positive cells and suggest that transient platelet sequestration might contribute to this initial drop in select animals. (B) Peripheral blood mononuclear cells were doubly stained with PE conjugated MWReg30 and DyLight 488 conjugated 2A-2HC. MWReg30 positive cells with low forward scatter and increased side scatter were gated as platelets. The histogram shows staining of the gated MWReg30 positive platelet population with 2A-2HC (dark, no fill) or isotype control (light, gray fill).
