# Supplementary material for: Fully recombinant IgG2a Fc multimers (stradomers) effectively treat collagen-induced arthritis and prevent idiopathic thrombocytopenic purpura in mice
Source: Arthritis Res Ther. 2012 Aug 20;14(4):R192. doi: 10.1186/ar4024 (PMC3580588; doi:10.1186/ar4024)
Supplement: Additional file 3 — Figure S3, 2A-2HC does not prevent graft-versus-host disease. This figure demonstrates that 2A-2HC cannot prevent T-cell-mediated graft-versus-host disease in a murine model. [file ar4024-S3.PPT]

## Slide 1
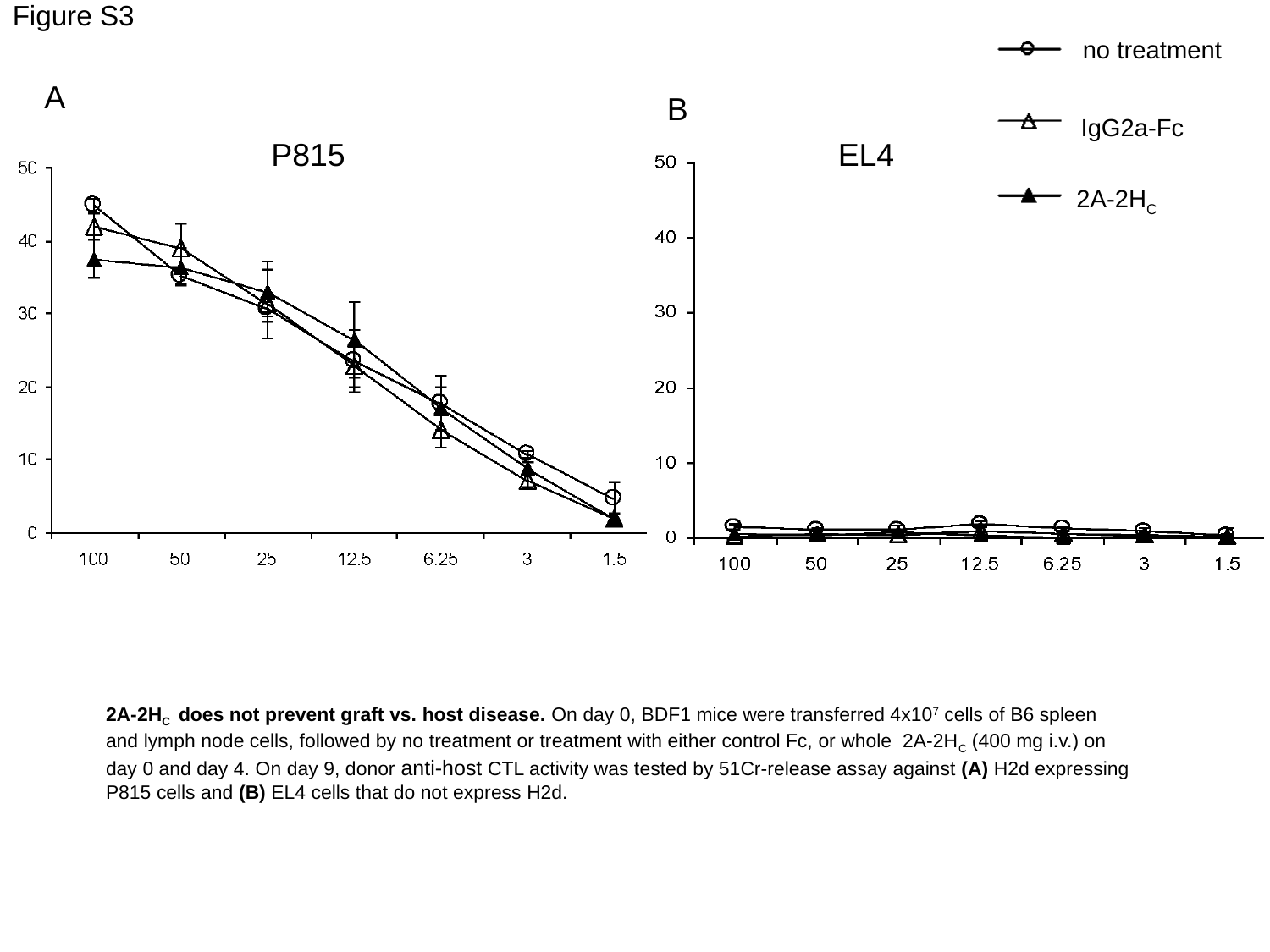

Figure S3
no treatment
A
B
IgG2a-Fc
P815
EL4
2A-2HC
2A-2HC does not prevent graft vs. host disease. On day 0, BDF1 mice were transferred 4x10^7^ cells of B6 spleen and lymph node cells, followed by no treatment or treatment with either control Fc, or whole 2A-2HC (400 mg i.v.) on day 0 and day 4. On day 9, donor anti-host CTL activity was tested by 51Cr-release assay against (A) H2d expressing P815 cells and (B) EL4 cells that do not express H2d.
