# Supplementary material for: Fully recombinant IgG2a Fc multimers (stradomers) effectively treat collagen-induced arthritis and prevent idiopathic thrombocytopenic purpura in mice
Source: Arthritis Res Ther. 2012 Aug 20;14(4):R192. doi: 10.1186/ar4024 (PMC3580588; doi:10.1186/ar4024)
Supplement: Additional file 4 — Figure S4, The FcγRIIb-/- and "wild type" C57/BL6 have differing baseline platelet and red blood cell counts. This figure demonstrates that FcγRIIb-/- and "wild type" C57/BL6 have differing baseline platelet counts, which could potentially affect the dynamic range of ITP assay in FcγRIIb-/- mice. [file ar4024-S4.PPT]

## Slide 1
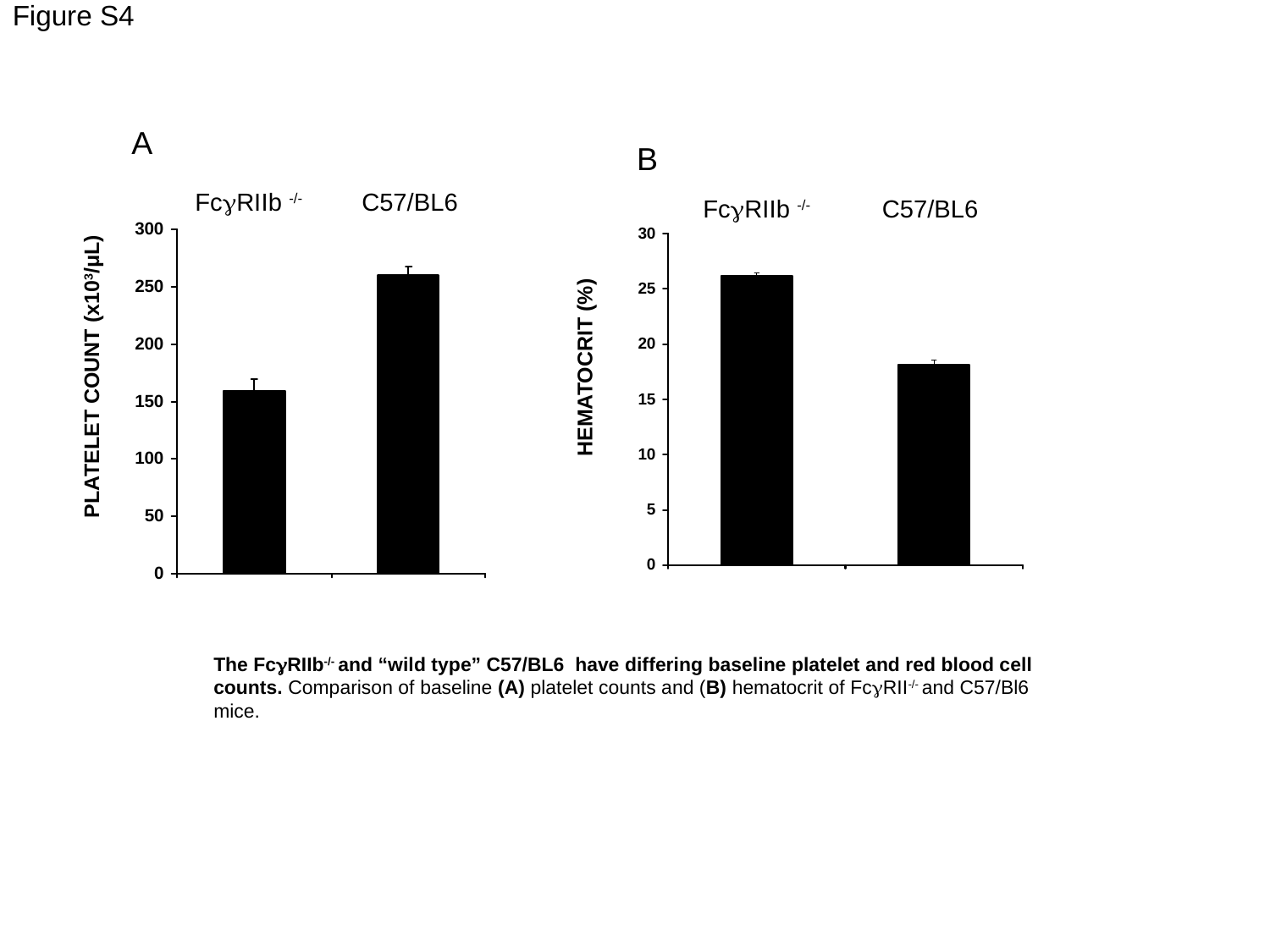

Figure S4
A
B
FcγRIIb -/-
C57/BL6
FcγRIIb -/-
C57/BL6
PLATELET COUNT (x103/μL)
HEMATOCRIT (%)
The FcγRIIb-/- and “wild type” C57/BL6 have differing baseline platelet and red blood cell counts. Comparison of baseline (A) platelet counts and (B) hematocrit of FcγRII-/- and C57/Bl6 mice.
